# A retrospective study investigating the rate of HER2 discordance between primary breast carcinoma and locoregional or metastatic disease

**DOI:** 10.1186/1471-2407-12-555

**Published:** 2012-11-24

**Authors:** Arlene Chan, Adrienne Morey, Belinda Brown, Diana Hastrich, Peter Willsher, David Ingram

**Affiliations:** 1 Mount Hospital, Perth, WA, 6000, Australia; 2 Sydpath, St Vincent’s Hospital, New South Wales, Australia

**Keywords:** HER2, Metastatic breast cancer, Discordance

## Abstract

**Background:**

Overall survival of HER2 positive metastatic breast cancer patients has been significantly improved with inclusion of trastuzumab to chemotherapy. Several studies have demonstrated discordant HER2 status in the primary and metastatic tumour. However, rates of discordance vary considerably in published reports.

**Methods:**

Information collected prospectively was analysed for all patients seen from 1999 to 2009 with primary breast cancer and who had biopsy of a local or distant recurrence. Patients were included if adequate tissue was available from both paired samples. Recurrent samples included fine needle aspirations, core and excisional biopsies. HER2 status in all paired samples was assessed by in-situ hybridisation by a single pathologist in a national reference laboratory. This was compared with HER2 immunohistochemistry results provided in the course of routine diagnosis at regional laboratories.

**Results:**

In total, 157 patients with recurrent (n = 137; 87.3%) or synchronous primary and metastatic (n = 20; 12.7%) breast cancer had biopsy of the metastatic site. The study population comprised of 116 patients with adequate tissue in both primary and metastasis. The concordance between HER2 status of the paired samples by local immunohistochemistry testing and central in-situ hybridization were 78% and 99%, respectively. Only one patient demonstrated HER2 discordance – primary lesion was positive whilst a metastatic site was negative.

**Conclusions:**

This single institution study demonstrated a low rate of HER2 discordance between primary and recurrent breast cancer as assessed by in-situ hybridisation. This contrasts to results reported by others, which may be explained by differences in study methodology, definition of recurrent disease samples and generally small numbers of patients assessed. Despite the current findings, the decision to obtain metastatic tissue for evaluation is influenced by other factors. These include disease-free interval, which may raise the possibility of a new malignancy and the accuracy of initial HER2 assessment of the primary tumour.

## Background

Optimal management of metastatic breast cancer requires accurate identification of the biological characteristics of the recurrent disease. In human epidermal growth factor receptor 2 (HER2) positive metastatic breast cancer, the clinical benefit of trastuzumab-based therapy is well established when compared with chemotherapy alone [1,2].

Further, it is established that the benefit of anti-HER2 therapy is largely achieved in those patients whose tumours are confirmed as being positive, either by 3+ HER2 protein expression on immunohistochemistry (IHC) or gene amplification by in-situ hybridization (ISH).

Retrospective studies have suggested that there may be clinically significant discordance between HER2 receptor status when comparing primary with recurrent/metastatic breast cancer of up to 42% [3-5]. Studies employing IHC have generally found higher discordance rates than those employing in situ hybridization, suggesting methodological issues may play a role in apparent discordance. Other factors, which may influence the rates of discordance between paired samples, include whether the same method of HER2 assessment is used for the primary and recurrent specimens [6,7].

To enable optimal management of this patient group, it is important to understand if the reported incidence of change in HER2 status of primary and metastatic breast cancer is real or an artefact of testing methodology. In the absence of definitive studies which use uniform methodology in the assessment of discordance between primary and recurrent breast cancer, retrospective single institution reports may provide some understanding of the significance of this occurrence.

The current study was undertaken to assess for the incidence of HER2 status of both primary and metastatic recurrence in patients from a single institution assessed in a high volume reference laboratory using uniform methodology, namely in-situ hybridization.

## Methods

### Study design

This is a retrospective, single center study, aimed to investigate the rate of HER2 neu discordance between primary breast carcinoma and locoregional or metastatic disease in patients seen by a single clinician at the Mount Hospital between 1999 and 2009.

Patient personal details were non-identifiable and all patients had provided written consent to their medical information being used for research purposes. The study was approved by the Mount Hospital Ethics and Research Committee and conducted in accordance with the Helsinki declaration.

### Study population

The study population comprised those patients who had adequate tissue available from paired primary and recurrent tumour samples for assessment of HER2 amplification. Patients who presented with primary breast cancer and synchronous metastatic disease who underwent biopsy of the metastatic lesion were also included. Patient demography, local laboratory determination of breast cancer pathological characteristics, management of primary and recurrent disease and follow-up information had been recorded prospectively over time in a secure database. Although the analysis was conducted retrospectively, source verification of entered data was possible given the nature of data collection. Primary breast cancer tissue sections were obtained from formalin-fixed paraffin embedded blocks and recurrent tumour samples were collected mainly as core biopsies or cell blocks prepared from centrifuged fine needle aspirations.

### HER2 assessment

HER2 status was assessed on paraffin sections by either single probe silver in situ hybridization (SISH: Ventana Inform HER2 assay) on Ventana XT automated stainer, or fluorescence in situ hybridization (FISH: Vysis/Abbott PathVysion HER2/cep17 dual colour assay). Overnight hybridization was employed in both assays. FISH was employed as either the primary assay in cases received for testing prior to 2006, or as a confirmatory assay in cases received after 2006 with non-diagnostic or equivocal SISH results. A positive FISH result was classified as a HER2/cep17 ratio >2.2 (high level amplified = ratio >4), and a positive single probe SISH result was classified as HER2 copy number >6 (6–10 low level amplified; >10 high level amplified). A negative polysomic result was defined as having mean HER2 copies >2.5 but < 4(diploid <2.5). Cases with 4–6 mean copies on single probe SISH were regarded as equivocal and re-assessed by FISH. All cases were scored by a single pathologist (AM), blinded as to the status of the paired sample.

### Statistical consideration

The agreement between the HER2 gene amplification status of the primary and recurrent lesion was assessed using a kappa test. All other variables are reported as a proportion of the eligible population where HER2 amplification status was possible on paired tumour samples.

## Results

### Patient characteristics

Over the 10 years study period, 157 women with recurrent (n = 137; 87.3%) or synchronous primary and de novo metastatic (n = 20; 12.7%) breast cancer underwent biopsy of the recurrence or metastatic site, respectively. Forty-one patients were excluded from this study due to insufficient tissue being available for central analysis; thus 116 patients constitute the study population. Thirty-six of the study patients (31%) were HER2 positive (3+) by local IHC testing of the primary tumour. Patient and tumour characteristics of the study population and those who had a recurrence biopsy but were ineligible are shown in Table 1. Patients in the study group were more likely to have had a recent breast cancer diagnosis with less than a 2-year interval between the paired biopsies. Of the 102 patients in the study population who presented with early breast cancer, the majority of tumours were invasive ductal (84.6%), grade 2 or 3 (93.1%) or associated with positive lymph nodes (80.2%) (Table 2). Eighty percent of patients received adjuvant chemotherapy with 10 patients receiving adjuvant trastuzumab in the context of a clinical trial.

**Table 1:** Patient and breast cancer characteristics

	**Study population**	**Ineligible population**	**p value**
	**n=116**	**n=41**	
	**Number (%)**	**Number (%)**	
Age at diagnosis			
Median (yrs)	50	50	
Range	(31–85)	(34–73)	
Year breast cancer diagnosis			
Pre - 2000	26 (22)	20 (49)	
2000–2001	19 (16)	6 (15)	0.01
2002–2003	29 (25)	3 (8)	
2004–2005	21 (18)	9 (23)	
2006–2007	16 (14)	2 (5)	
2008-2009	5 (4)	1 (3)	
Disease interval to biopsy (yrs)			
0 (metastatic at diagnosis)	9 (8)	5 (12)	
0.5 – 2y	29 (25)	2 (5)	0.02
2.1 – 5	44 (38)	14 (34)	
>5y	34 (29)	20 (49)	
Type of biopsy			
Local recurrence site	40 (34)	7 (17)	0.05
Distant recurrence site	76 (66)	34 (83)	
Site of recurrent or metastatic biopsy			
Breast	24 (20)	3 (7)	
Lymph nodes	20 (17)	10 (24)	
Chest wall / Skin	18 (16)	4 (10)	0.13
Bone	14 (12)	9 (22)	
Liver	9 (8)	5 (12)	
Brain	9 (8)	2 (5)	
Lung	7 (6)	0	
Others	15 (13)	8 (20)	
Type of tissue biopsy			
Fine needle aspiration	34 (29)	13 (32)	0.84
Core / Excisional biopsy	82 (71)	28 (68)	

**Table 2:** Characteristics of breast primary in study population

	**Number of patients (%)**
Stage at diagnosis	
1	20 (17.2)
2	52 (44.8)
3	30 (25.9)
4	14 (12.2)
Grade	
1	8 (6.9)
2	51 (44)
3	57 (49.1)
Nodal status	
Negative	23 (19.8)
Positive	93 (80.2)
HR status	
ER and/or PR positive	74 (63.8)
ER and PR negative	42 (36.2)
HER2 status	
Negative	79 (68.1%)
Positive	37 (31.8%)
Neoadjuvant or Adjuvant treatment	
Nil or non-compliant	14 (13.7)
Endocrine only	6 (5.9)
Non-anthracycline chemotherapy	11 (10.8)
Anthracycline-base chemotherapy	38 (37.3)
Anthracycline and taxane	29 (28.4)
Taxane only	4 (3.9)
Adjuvant trastuzumab	10 (8.6)
Disease-free interval, median (range)	36.3 (26.2 – 135)

At the time of disease recurrence, 29 patients received HER2 targeted treatment in the first-line metastatic setting. Median duration of HER2 targeted treatment in these patients was 9.4 months (3.6 – 68.6), with a slightly shorter duration of treatment exposure in those patients who had received adjuvant trastuzumab compared to those who had not (median 8.3 months vs 9.4 months, respectively).

### HER2 Concordance rates

Local evaluation of the primary and recurrent lesion by IHC is shown in Table 3, with 78% concordance between the paired samples when categorising as negative (0 or 1+), inconclusive (2+) or positive (3+). In contrast, central analysis of paired samples demonstrated 99% concordance between the primary and paired recurrence biopsy with respect to HER2 amplification status as assessed by ISH, when status was classified as either positive or negative (Table 4). The kappa score for paired samples as assessed by immunohistochemistry was 0.616, which demonstrates good agreement. For in situ hybridisation, the kappa score of 0.979 (95% CI 0.939 – 1.02) indicates very good agreement.

**Table 3:** HER2 status of primary and matched recurrent lesion by immunohistochemistry*

**PRIMARY**	**RECURRENCE**		
	**HER2 negative**	**HER2 inconclusive**	**HER2 positive**
HER2 negative	50	8	2
HER2 inconclusive	4	5	2
HER2 positive	2	3	22
Total	56	16	26

*Eighteen patients, where immunohistochemistry was not performed by the local laboratory on primary or recurrence, were excluded.

**Table 4:** HER2 status of primary and matched recurrent lesion by in-situ hybridization

**PRIMARY**	**RECURRENCE**			
	**Negative**	**Negative Polysomic**	**Low Amplified**	**High Amplified**
Negative	53	11	0	0
Negative Polysomic	9	6	0	0
Low Amplified	0	0 (2*)	2	5
High Amplified	1	0	1	26

The 2* cases which did not contain breast malignancy upon central review were excluded.

The only patient to demonstrate apparent genuine change in status was a 78 yr old woman who was diagnosed with HER2 amplified left breast cancer (HER2/cep17 FISH ratio = 4.1) and subsequently developed metastatic recurrence in the bones, 34 months later. Biopsy of the sacrum demonstrated metastatic breast cancer and the patient was commenced on trastuzumab-based treatment, in a clinical trial setting. Following 28 months of objective disease control, she developed progressive bone disease and locoregional recurrence in the left breast. A biopsy of the breast lesion demonstrated a mean HER2 gene copy number of 2.97 consistent with polysomy. She continued treatment with trastuzumab-based therapy with the addition of endocrine treatment and continues to have responsive disease to the present time (64 months). Nine patients who had received adjuvant trastuzumab-based therapy developed recurrent disease; in all cases, the metastatic lesion remained concordant for HER2 positivity by in situ hybridisation.

Two patients with HER2 amplified primary breast cancers had apparent negative HER2 ISH status in metastatic deposits (brain and pleural fluid respectively) at initial blinded assessment. Subsequent histological examination and IHC on the brain lesion confirmed it was an unrelated primitive ectodermal primary brain tumour. Additional IHC on the pleural fluid cell block confirmed the presence of reactive mesothelial cells only. These cases were thus retrospectively classified as “ineligible” due to the absence of assessable metastatic breast cancer, but have been included in Table 3 for completeness.

## Discussion

Several publications have reported discordance in the HER2 status between primary breast cancer and metastatic disease. The alteration in the HER2 status from positive to negative has ranged from 2% to 42%. Changes in the HER2 status in the opposite direction has also been reported, with some authors reporting rates of up to 37%. The variation in reported results may relate to several factors. These include the method used to evaluate HER2 status in the paired specimens, the definition of “metastatic” tissue to which the primary HER2 status is compared, whether HER2 status is evaluated through the detection of gene amplification in tissue sections or as circulating HER2 protein levels, and whether anti-HER2 treatment is administered to patients prior to obtaining the second specimen.

Our study underscores the difficulties in assessing paired primary and recurrent tumour specimens when analysis is performed in a retrospective fashion with 26% of specimens having insufficient material available for in situ hybridisation. Although the majority of patients had core or excisional biopsies of the recurrent lesion, there was still inadequate tissue available for central assessment in a significant proportion of patients. Giotta et al. demonstrated in a small study of 20 patients that it was feasible to perform in situ hybridisation on cytological specimens obtained from 21–23 gauge needle biopsies [8]. They reported a HER2 discordance rate of 10% with one negative primary lesion becoming amplified in a subsequent lung metastasis; and one HER2 amplified primary lesion being associated with loss of amplification in a liver metastasis.

There have been conflicting results in studies that have assessed HER2 status with a combination of immunohistochemistry and in situ hybridisation of the primary and metastatic lesion (Table 5). The HercepTest™ (Dako, Glostrup, Denmark) or FISH were used in a study of 100 paired primary and metastatic samples, where a discordance rate of 6% was found, with all 6 cases showing HER2 overexpression in the metastatic lesion compared to the HER2-negative primary tumour [9]. Metastatic samples included biopsies from bone, soft tissue and viscera. Fluorescent in situ hybridisation was only possible in 68 paired samples and there were 5 discordant cases (7%); 3 metastases gaining amplification vs a non-amplified primary, and 2 metastases becoming non-amplified. The study identified 11% of cases which were negative on immunohistochemistry but confirmed as positive on in situ hybridisation [9]. Thus, the authors concluded that re-biopsy of a metastasis for the purpose of confirming HER2 status of the recurrence was not supported with the exception of primary tumours assessed as HER2 negative on immunohistochemistry alone, where biopsy of a recurrence for analysis by in situ hybridisation was indicated. Recently, Niikura et al. identified forty-three (24%) of the 182 patients with HER2 positive primary tumors as having metastatic tumors which were HER2 negative [10]. However the authors accepted both IHC3+ and ISH+ results as indicators of positive primary status without central review of these specimens for the purposes of their study [10]. The majority of the patients had been treated with adjuvant chemotherapy and trastuzumab. They reported significantly higher rates of HER2 discordance in those patients who had received adjuvant chemotherapy compared to those who had not. These authors argued strongly for re-biopsy of metastatic lesions to accurately plan management [10]. In the same issue of the journal, Amir et al. reported their prospective study of patients presenting with imaging suggestive of metastatic disease or who were experiencing progression while receiving palliative systemic treatment [11]. The authors demonstrated discordance in HER2 status (as assessed by FISH) in 9.6% of 83 assessable patients (gain in 6/73, loss in 2/10); and concluded that biopsy of metastases was feasible and led to change in systemic therapy in 14% of patients [11].

**Table 5:** Summary of studies reporting HER2 status in primary breast cancer and metastases

**Author**	**Patient numbers**	**“Gain” in HER2**	**“Loss” of HER2**
Giotta [8]	20	5%	5%
Gancberg [9]:			
Immunohistochemistry	100	6%	-
In situ hybridisation	68	4%	3%
Nikura [10]	182	-	24%
Amir [11]	83	8% (6/73)	20% (2/10)
Gong [12]	60	1.6%	1.6%
Tapia [13]	105	1.9%	0.9%
Fabi [14]	137	8.7%	1.5%
Simon [15]	122	2.2%	6.5%
Lindstrom [16]	76	6.5%	10.5%

Gong et al. compared primary tumour with loco-regional and distant recurrence in 43 and 17 patients, respectively [12]. Thirty-two patients had received chemotherapy in the period between the primary and recurrence biopsies. It was possible to examine HER2 status by fluorescent in situ hybridisation on paraffin-embedded tissue or fine needle aspirates. All but 2 of the 60 tumours were concordant; one case demonstrated HER2 negative primary from one of three multifocal lesions, whilst the axillary nodal metastasis was positive. The second case showed amplification in the primary but not in the liver metastasis. Therefore HER2 status was reliably assessed in the primary with 97% concordance and it was considered that the HER2 status remained stable during the metastatic process [12].

Tapia et al. reported an initial discordance rate of 7.6% in 105 patients whose primary and metastatic lesions had undergone HER2 evaluation by FISH on primary histological and metastatic cytological specimens [13]. The 8 discordant cases were re-evaluated by FISH and 5 of the cases were found to be concordant. Reasons for the discordant initial assessment included interpretational error with the HER2/reference ratio being close to 2.0 in three patients, and re-evaluation identified the presence of scanty amplified malignant cells which had been initially overlooked in two patients [13]. The authors concluded that HER2 gene status remains highly conserved between primary and metastatic disease with a final concordance rate of 97.1% in their sample [13]. In contrast to these studies, a recent report by Fabi et al. in 137 patients diagnosed between 1999 and 2006 demonstrated a discordance rate of 10%, 12 primary lesions being HER2 negative whilst the paired metastasis was positive; and 2 patients with a change in the HER2 status in the opposite direction [14]. The strength of this study was uniform use of silver in situ hybridisation (SISH) for assessment of the paired samples. A further finding in this group was the significant increase in gene copy number in the metastases of tumours that were amplified in the primary lesion as defined by SISH [14].

Simon et al. evaluated tissue microarrays of primary tumour and lymph node positive metastases, where the HER2 status was assessable in 125 patients. In this patient group, a discordance rate of 7.2% (9 patients) was found overall. However, only 2 patients had nodal metastases, which were uniformly discordant to the primary tumour. The remaining patients had nodal metastases in which some showed HER2 concordance with the primary tumour, illustrating the heterogeneity that may exist [15]. A recent Swedish study demonstrated 14.5% discordance between the primary and metastatic lesion; although this frequency increased to 50% when considering those tumours, which converted to or maintained oestrogen negativity in the metastases [16].

Several groups have assessed the impact of systemic treatment with or without HER2 targeted therapy on subsequent tumour HER2 status. Results on 142 HER2-positive patients (defined as IHC 3+ or amplification on ISH) treated with neoadjuvant anthracyclines, taxanes and trastuzumab over the period 2004–2007 were reported from the MD Anderson Cancer Centre. In 25 patients with sufficient residual invasive tumour, comparison of HER2 status by FISH was performed [17]. Eight patients (32%) had residual disease which was HER2 negative and at median follow-up of 37 months, this group of patients had significantly inferior relapse-free survival compared to those patients whose residual disease remained HER2 positive [17]. These results contrast with a study, which utilised immunohistochemistry to evaluate HER2 status in residual disease in the breasts of 15 patients receiving anthracycline-based neoadjuvant therapy (trastuzumab was not given) and 44 patients with metastatic disease who underwent surgical resection or biopsy of localised liver or lung metastases [18]. In both patient groups, patients who had HER2 positive disease at baseline evaluation were found to have identical HER2 over-expression in the residual disease (11 of 13 breast specimens; and 9 of 9 metastases).

No cases of heterogeneous HER2 amplification were detected in our study cohort, although two cases were noted to be heterogeneous with respect to the presence of polysomy. The incidence of heterogeneity of HER2 status in breast cancer (as determined by ISH) is variably estimated at up to 11%, and this may underlie some of the cases of “genuine” HER2 status change, reflecting outgrowth of an undetected clone [19]. Re-assessing HER2 status in metastatic deposits of any case exhibiting heterogeneity in the primary tumour would appear to be warranted.

## Conclusion

In conclusion, the present study is one of the largest studies where paired primary and recurrence tissue samples were available for centralised ISH analysis. The limitations of a retrospective review does not permit the results to impact on current clinical practice, but our study does provide further evidence confirming a very low incidence of change in the HER2 status between primary and recurrent breast cancer when a uniform and reliable methodology is employed. To avoid misinterpretation of discordance rates between paired samples over time, our study would indicate that it is important to use the same method of HER2 assessment on the primary and recurrence specimens. Further we have demonstrated that in situ hybridisation is more accurate than immunohistochemistry and less susceptible to sample processing variables.

It is not possible to fully explain the variation in reporting of HER2 discordance rates in the literature, but factors include small numbers (<100) of patients evaluated, including those where the actual number of paired samples studies were less than the entire cohort. The use of a combination of immunohistochemistry and in situ hybridisation or other non-standard methods of evaluation (such as automated subcellular localization and quantification of protein expression multiplex ligation-dependent probe amplification) may also influence the interpretation of results.

Although the current study did not demonstrate discordance in HER2 status such that management of recurrent disease was altered, there exists the possibility that apparent recurrent breast cancer may be a new primary malignancy and therefore factors such as long disease-free interval, atypical radiological appearance and clinical judgement as to the baseline breast cancer risk and suspicion of recurrence needs to be considered.

## Abbreviations

HER2: Human epidermal growth factor receptor 2; IHC: Immunohistochemistry; ISH: In situ hybridization; FISH: Fluorescent in situ hybridization; SISH: Silver in situ hybridization; cep17: Chromosome 17 centromere; DTC: Disseminated tumour cells; CTC: Circulating tumour cells.

## Competing interests

AC has received honoraria and research grant for educational speaking engagements / consultancy advice and investigator-initiated study, respectively. AM, BB, DH, PW, DI have no conflicts of interest to declare.

## Authors’ contribution

AC designed study, performed the clinical assessments, analysed the data and prepared the manuscript. AM, BB, performed the laboratory experiments and analysed the data and prepared the methodology section of the manuscript. DH, PW, DI participated in the study design, contributed to clinical data collection and data analysis. All authors contributed to and approved the final manuscript.

## Pre-publication history

The pre-publication history for this paper can be accessed here:

http://www.biomedcentral.com/1471-2407/12/555/prepub
